# Metallic conduction induced by direct anion site doping in layered SnSe_**2**_

**DOI:** 10.1038/srep19733

**Published:** 2016-01-21

**Authors:** Sang Il Kim, Sungwoo Hwang, Se Yun Kim, Woo-Jin Lee, Doh Won Jung, Kyoung-Seok Moon, Hee Jung Park, Young-Jin Cho, Yong-Hee Cho, Jung-Hwa Kim, Dong-Jin Yun, Kyu Hyoung Lee, In-taek Han, Kimoon Lee, Yoonchul Sohn

**Affiliations:** 1 Department of Materials Science and Engineering, The University of Seoul,, Seoul 130-743, South Korea; 2 Materials R&D Center, Samsung Advanced Institute of Technology, Suwon 443-370, Republic of Korea; 3 Platform Technology Laboratory, Samsung Advanced Institute of Technology, Suwon 443-370, Republic of Korea; 4 Department of Advanced Materials Engineering, Daejeon University, Daejeon 300-716, Republic of Korea; 5 Department of Nano Applied Engineering, Kangwon National University, Chooncheon 200-701, Republic of Korea; 6 Department of Physics, Kunsan National University, Gunsan 573-701, Republic of Korea

## Abstract

The emergence of metallic conduction in layered dichalcogenide semiconductor materials by chemical doping is one of key issues for two-dimensional (2D) materials engineering. At present, doping methods for layered dichalcogenide materials have been limited to an ion intercalation between layer units or electrostatic carrier doping by electrical bias owing to the absence of appropriate substitutional dopant for increasing the carrier concentration. Here, we report the occurrence of metallic conduction in the layered dichalcogenide of SnSe_2_ by the direct Se-site doping with Cl as a shallow electron donor. The total carrier concentration up to ~10^20^ cm^−3^ is achieved by Cl substitutional doping, resulting in the improved conductivity value of ~170 S·cm^−1^ from ~1.7 S·cm^−1^ for non-doped SnSe_2_. When the carrier concentration exceeds ~10^19^ cm^−3^, the conduction mechanism is changed from hopping to degenerate conduction, exhibiting metal-insulator transition behavior. Detailed band structure calculation reveals that the hybridized *s-p* orbital from Sn 5*s* and Se 4*p* states is responsible for the degenerate metallic conduction in electron-doped SnSe_2_.

Two dimensional (2D) materials have recently been intensively investigated due to its unique electronic and/or optical properties^1^,^2^,^3^,^4^,^5^,^6^,^7^. Since the remarkable properties of graphene have initiated the research, other 2D materials with layered structure are also emerged as new counterparts to exhibit striking physical properties under the 2D confined systems^1^,^2^,^3^. Layered dichalcogenides are one of the representative materials drawing great attention from the fact that they can be easily prepared as a form of high quality nanosheet by mechanical and/or chemical exfoliation techniques as graphene did^2^,^4^.

As a practical engineering method to modulate 2D material properties, doping is an important technique especially for controlling electrical characteristics. Until now, the doping methods for 2D materials have been tried mainly by electrostatic carrier doping introduced by the electrical bias through an dielectric barrier^5^,^6^, or insertion of interstitial intercalants between the layer units which are weakly bonded each other by Van der Waals interaction^7^,^8^,^9^. In particular, for the case of layered dichalcogenides, striking carrier transport characteristics can be provoked by effective carrier doping^5^,^6^,^8^,^9^, but direct substitutional doping has been rarely reported due to the absence of adequate substitutional dopant to the best of our knowledge^10^.

In this study, we report the emergence of metallic conduction in layered dichalcogenide of SnSe_2_ achieved by direct Se-site doping with Cl atoms. Substitutional Cl dopant on Se-site worked well as a shallow electron donor to increase carrier concentration (*n*_*e*_) up to ~10^20 ^cm^−3^, and the transition from semiconductor to metallic state was observed when *n*_*e*_ exceeded critical limit (~10^19 ^cm^−3^). Based on the electronic structure calculation, it was clarified that the hybridization between largely spread Sn 5*s* and Se 4*p* orbital is the origin of degenerate conduction in Cl-doped SnSe_2_.

## Results and Discussion

**Figure 1:**
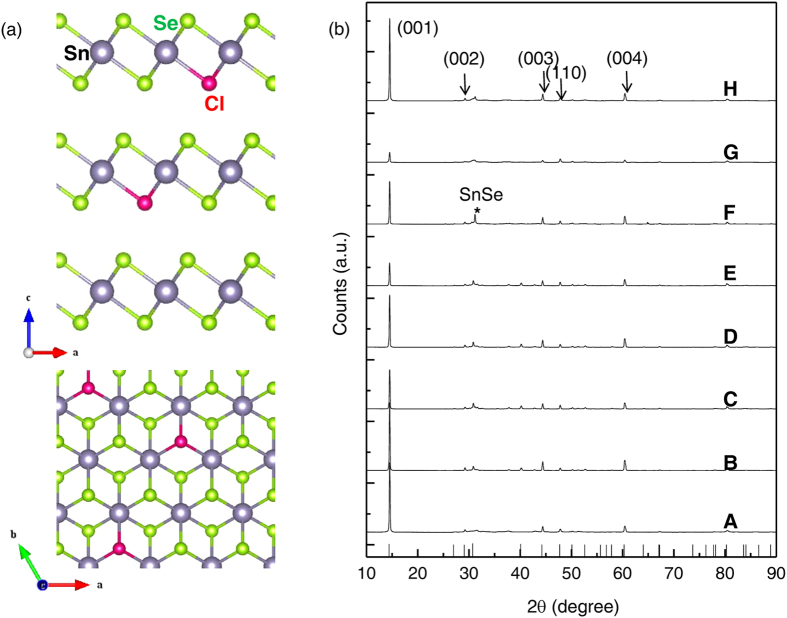
Structural characterizations of Cl-doped SnSe_2_. (**a**) Schematics of crystal structure of Cl-doped SnSe_2_ along b- (upper) and c-axis (lower). (**b**) The patterns of powder X-ray diffraction from various amount of Cl-doped SnSe_2_ samples [A: undoped (n_e_ = 7.2 × 10^17 ^cm^−3^), B–H: doped samples with increasing Cl doping ratio from B (n_e_ = 5.1 × 10^18 ^cm^−3^) to H (n_e_ = 8.6 × 10^19 ^cm^−3^)]. Reference peak positions of SnSe_2_ are displayed at the bottom of the graph. Representative lattice planes are indicated and SnSe impurity phase is marked with arrows and asterisk, respectively.

Figure 1(a) schematically illustrates the crystalline structure of SnSe_2_ as characterized by the powder X-ray diffraction (PXRD) results in figure 1(b). SnSe_2_ has a hexagonal layered structure (*P-3m1* space group) with lattice parameters of a = 3.81 Å and c = 6.13 Å, which agree well with those reported in literature^11^. Fig. 1(b) shows the PXRD results with increasing Cl dopant ratio introduced during SnSe_2_ synthesis. The doped samples were named from A to H with increasing dopant content as listed in Table 1. No observable impurity compound associated with Cl dopant was detected except for small amount of SnSe phase in the sample F. The lattice parameters of the samples were calculated from the PXRD patterns and also listed in Table 1. They are almost identical within 0.1% variation for all samples. Similar lattice parameters of the samples are attributed to the fact that Se^2−^ (1.98 Å) has similar ionic radius to Cl^−^ (1.81 Å), supporting that Cl dopants are not located on the inter-layer space as intercalants, but on the Se site as substitutional atoms for Se^2−^ to minimize the lattice deformation^12^. The theoretical prediction for formation energies of probable defect states also indicates that Cl substitution to Se-site is the most stable compared to other defect candidates. (Figure S1 in the supplementary information).

**Figure 2:**
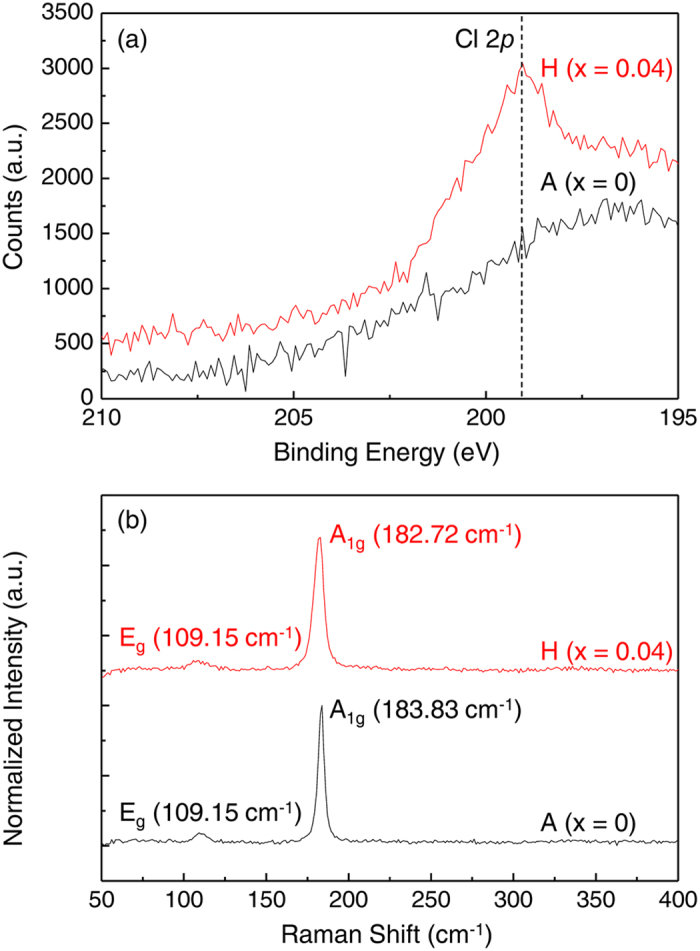
XPS and Raman spectroscopy. **(a)** X-ray photoemission and (**b**) Raman spectroscopy results for sample A (x = 0, non-doped SnSe_2_) and sample H (x = 0.04) Dashed line in Fig. 2(a) remarks the characteristic Cl^-^ peak position.

To more strongly reveal the Cl dopant substitution on Se-site in SnSe_2_ samples, we performed X-ray photoemission spectroscopy (XPS) as well as Raman spectroscopy with non-doped (A of x = 0) and the most heavily doped sample (H of x = 0.04). As shown in figure 2(a), characteristic Cl 2*p* core level peak at 199.05 eV was observed with sample H while sample A did not show any Cl related one. From the fact that the measured core level corresponds well with Cl^–^ state which is almost identical to the Cl 2*p* position in the form of CdCl_2_^13^, it indicates that the Cl dopant exists as Cl^–^ state which could serve well as an electron donor if it substitutes on Se^2−^ site. Figure 2(b) displays the Raman spectroscopy results with the same samples. With sample H, we could observe not only the downshift but also the peak broadening occurred for A_1g_ mode compared to those from sample A^14^. It strongly evidences that Cl dopant well substitutes on Se-site as decreasing the force constant in SnSe_2_ as reported in other substitutionally doped materials^15^,^16^,^17^.

**Figure 3:**
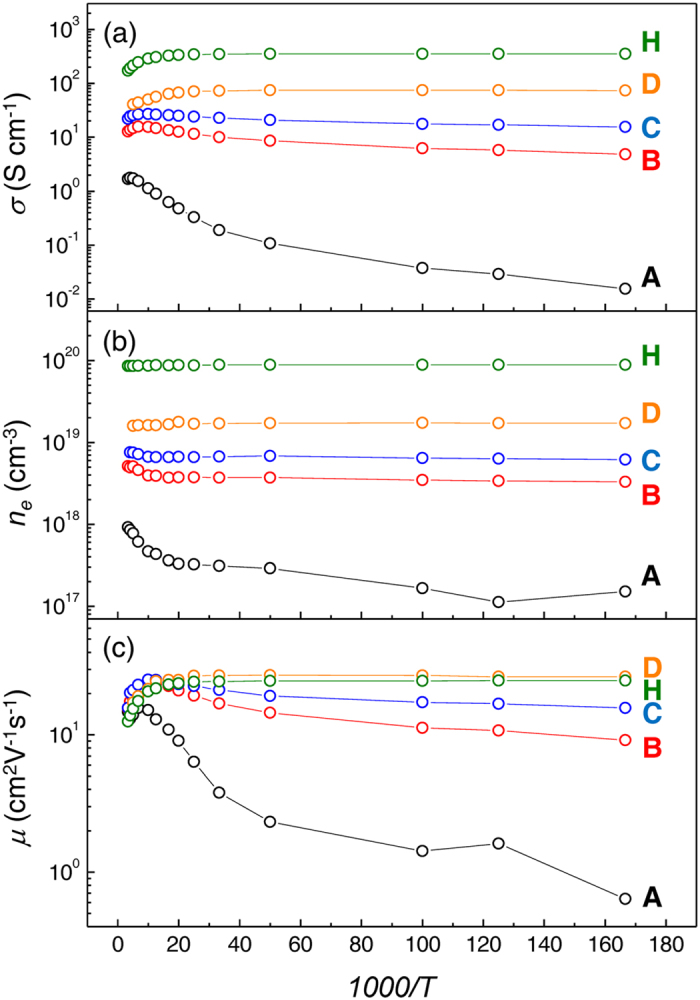
Electron transport properties of Cl-doped SnSe_2_. Temperature dependence of (**a**) electrical conductivity, (**b**) carrier concentration and (**c**) mobility of Cl-doped SnSe_2_ samples.

**Figure 4:**
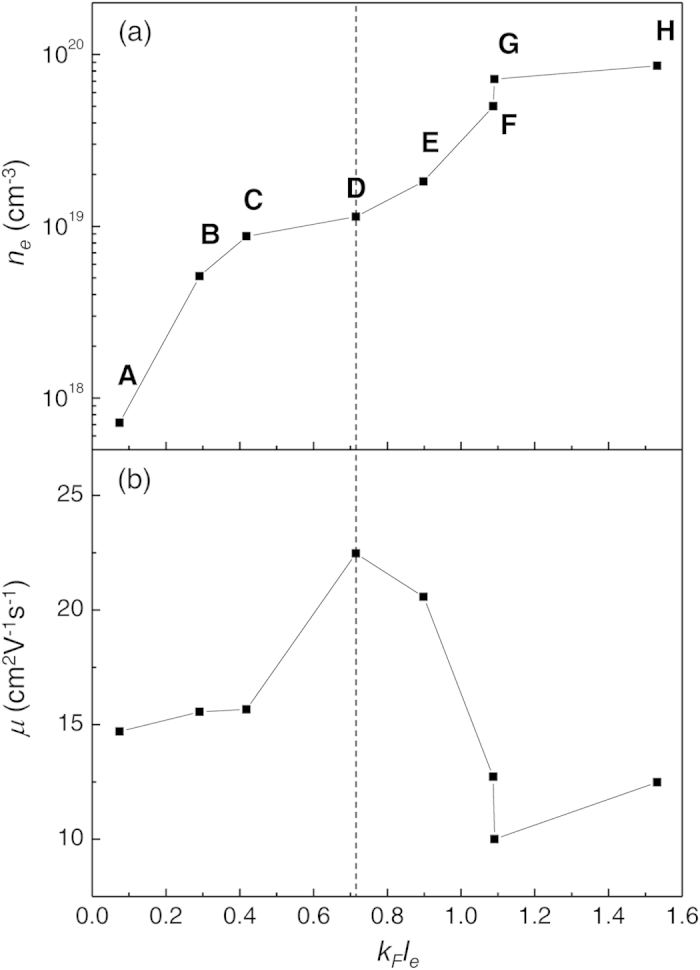
Disorder parameters of Cl-doped SnSe_2_. Disorder parameter (*k*_*F*_*l*_*e*_) dependences of (**a)** carrier concentration and (**b)** mobility of Cl-doped SnSe_2_ samples at 300 K. Dashed line points out the critical point [(*k*_*F*_*l*_*e*_)_*c*_ = 0.71] where metal-insulator transition occurs.

Figure 3(a–c) shows the temperature (*T* ) dependence of conductivity (σ), *n*_*e*_, and mobility (*μ*), respectively for Cl-doped SnSe_2_ samples, extracted from the measurement of four point-probe resistivity and Hall coefficient. As shown in Fig. 3(a), σ exhibits thermally activated behavior from A to C sample, while the trend changes to degenerate conduction for sample D to H. Hall-effect measurement reveals that all SnSe_2_ samples are n-type and their *n*_*e*_ value increases up to 8.6 × 10^19 ^cm^−3^ (H sample with nominal Cl content, x of 0.04) with the maximum σ value of ~170 S·cm^−1 ^at 300 K as Cl doping ratio increases as shown in Fig. 3(b). (The *n*_*e*_ values of the sample E, F and G are located in-between those of the sample D and H (refer to Fig. 4), showing same electrical behavior and removed for brevity). It proves that Cl dopants act as electron donors to SnSe_2_ and that Cl^−^ is successfully substituted on Se^2−^ site simultaneously supported by XPS and Raman results. (See Fig. 2) It should be noted that *n*_*e*_ becomes *T* independent when degenerate conduction occurs as is the case with sample D and H. It indicates that the Fermi level (*E*_*F*_) exceeds the conduction band edge of SnSe_2_ as *n*_*e*_ increases over 10^19 ^cm^−3^ 18. *T* dependence of *μ* was deduced from the relation of σ = *en*_*e*_*μ*, where *e* is the charge of electron and presented in Fig. 3(c)^19^. When *n*_*e*_ is less than 10^19 ^cm^−3^, *μ* is limited by lattice vibration scattering at high *T* region, while limiting scattering mechanism changes to ionized impurity scattering at low *T* region as also displayed in figure S2(b) in supplementary information^20^. (The transition point is observed at about 100 K.) With the samples with high *n*_*e*_ over 10^19 ^cm^−3^, *μ* value keeps increasing as *T* decreases and saturates at specific values, indicating that *μ* of the samples is governed dominantly by lattice vibration scattering in whole *T* region, as is the case with typical metals^19^. Experimental results of electrical transport strongly support the emergence of metallic states from layered SnSe_2_ semiconductor by Cl substitutional doping on Se-site.

To investigate the origin of semiconductor to metal transition in Cl-doped SnSe_2_, we estimate the disorder parameter, *k*_*F*_*l*_*e*_, based on the Ioffe-Regel criterion, which can be determined by^6^,^21^,^22^,^23^,


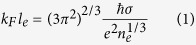


where *k*_*F*_ is Fermi wave number, *l*_*e*_ is electron mean free path, and *ħ* is Plank’s constant divided by 2π. In figure 4(a), the relation between *k*_*F*_*l*_*e*_ and *n*_*e*_ at 300K is plotted based on equation (1). From the fact that degenerate conduction occurs when *n*_*e*_ exceeds 1.13 × 10^19 ^cm^−3^ as observed in Fig. 3, we can obtain the critical value of (*k*_*F*_*l*_*e*_)_*c*_ as 0.71 where the metal-insulator transition occurs. (Note that the metal-insulator transition point can also be confirmed by estimating *w(0)* from the mathematical function of *w(T)* = *d(ln*σ*)/d(lnT)* as shown in figure S3^24^). As shown in figure 4(b), *μ* increases with increasing *k*_*F*_*l*_*e*_ when *k*_*F*_*l*_*e*_ < *(k*_*F*_*l*_*e*_)_*c*_, while it starts decreasing when *k*_*F*_*l*_*e*_ > *(k*_*F*_*l*_*e*_)_*c*_. As Pearson and Bardeen have proposed, donor ionization energy (*E*_*d*_) should decreases with increasing *n*_*e*_ for the doped semiconductor with a substitutional dopant atom due to the decrease of average potential energy of electrons^25^. *E*_*d*_ can be calculated from the slope of *ln(n*_*e*_) versus *1/T* plot (Refer to figure S2(a) in supplementary information) for the case of *k*_*F*_*l*_*e*_ < (*k*_*F*_*l*_*e*_)_*c*_ based on the relation below^19^,


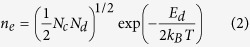


where *N*_*c*_ is the effective density of states at conduction band edge, *N*_*d*_ is donor concentration, and *k*_*B*_ is the Boltzmann constant. The values are estimated as 13.97, 6.95, and 3.77 meV for *n*_*e*_ of 7 × 10^17^, 5 × 10^18^, and 9 × 10^18 ^cm^−3^ at 300 K, respectively. It strongly suggests that the increase of *μ* with *n*_*e*_ increment (for *k*_*F*_*l*_*e*_ < (*k*_*F*_*l*_*e*_)_*c*_) mainly originates from enhanced *l*_*e*_ with reduced *E*_*d*_ which is deeply associated with trap energy levels of the scatterers in the semiconductor regime^25^. On the other hand, when *n*_*e*_ exceeds the critical value, (*k*_*F*_*l*_*e*_)_*c*_, Cl dopants starts to act as scattering centers, then *μ* starts decreasing due to ionized impurity scattering. As a consequence, it can be concluded that metallic transition of conduction mechanism for SnSe_2_ is primarily due to sufficient electron concentration and long electron mean free path achieved from shallow *E*_*d*_ level.

**Figure 5:**
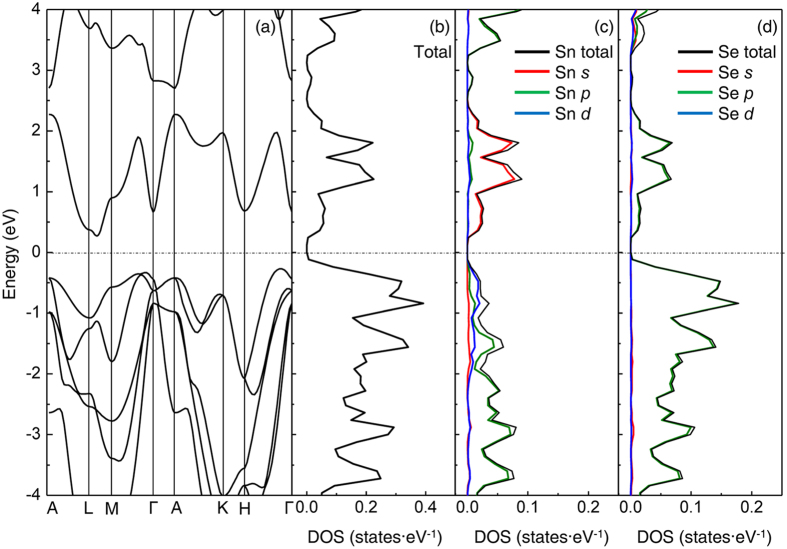
Electronic structure of SnSe_2_. **(a)** Calculated band structure of SnSe_2_. Fermi energy level is taken as origin. (**b–d**) Density of states profiles from total, Sn and Se contribution, respectively.

Figure 5(a)–(d) describe the calculated band structure and density of states (DOS) profiles of un-doped SnSe_2_ based on density functional theory (DFT). As shown in Fig. 5(a), *E*_*F*_ is set to be zero in the energy scale. The conduction band minimum (CBM) and valence band maximum (VBM) are located at L-M and H-Γ path, respectively, indicating the indirect band-gap semiconductor nature which agrees well with our experimental results for non-doped SnSe_2_. Since the structural parameters are almost identical regardless of Cl doping ratio as confirmed by PXRD results (see Table 1), it is reasonable to assume the rigid band behavior with raised *E*_*F*_ upon CBM for an electron-doped SnSe_2_ system. As *n*_*e*_ increases, *E*_*F*_ shifts upward until it meets CBM, and degenerate conduction should occur based on the CBM state, as are the case with typical heavily doped degenerate semiconductors^18^,^26^. As observed from the results of total and projected DOS in Fig. 5(b)–(d), the total DOS mainly consists of Sn 5*s* and Se 4*p* orbitals with similar contributions, indicating that the metallic conduction of electron-doped SnSe_2_ originates from the hybridized orbital states between Sn 5*s* and Se 4*p* orbital. It is worthwhile to note that (*k*_*F*_*l*_*e*_)_*c*_ value of 0.71 for electron-doped SnSe_2_ is close to those of conducting oxide materials showing largely spread orbital conduction such as In 5*s* ((*k*_*F*_*l*_*e*_)_*c*_ = 0.1 ~ 0.3 for In_2_O_3_, In-Ga-Zn-O and In-Zn-O), but much smaller than the reported value of other layered transition metal dichalcogenide. ((*k*_*F*_*l*_*e*_)_*c*_ = 2.5 for electron-doped MoS_2_)^6^,^22^,^23^. It strongly suggests that electron pathways in SnSe_2_ would be dispersed well, compared to localized electrons in other layered chalcogenide materials, due to effective overlap of delocalized large Sn 5*s* orbital as observed in In-based conducting oxide group. Therefore, metallic conduction of Cl-doped SnSe_2_ could be attributed to a large number of electrons supplied by efficient substitutional doping and their de-localization behavior originated from Sn 5*s* orbital.

## Conclusions

In summary, we successfully synthesized metallic layered SnSe_2_ from original semiconducting state by direct substitutional doping of Cl atoms. Doped Cl atoms on Se-site acted well as shallow donors to introduce high *n*_*e*_ up to ~10^20 ^cm^−3^, changing conduction mechanism to degenerate conduction from thermally activated conduction of undoped SnSe_2_. Detailed analysis of carrier transport mechanism and calculation of band structure confirm that metallic transport in electron-doped SnSe_2_ originates from highly dispersive Sn 5*s* orbital which is hybridized with Se 4*p* state. We anticipate that the engineering of conductivity from semiconducting to metallic states by direct chemical doping can be used as key technology for practical applications of 2D layered dichalcogenide materials, as has been proven for Si-based technology.

## Methods

### Sample synthesis

To synthesize polycrystalline SnSe_2_ samples doped with Cl, we mixed stoichiometric amount of Sn and anhydrous SnCl_2_ powders: (2−x)Sn + xSnCl_2 _→ 2SnCl_x_, and then melted the mixtures in fused silica tubes at 250 °C for 24 hrs. (Detailed nominal x’s are listed in Table 1.) After the first reaction was finished, stoichiometric Se grains were added to synthesize the final compound of Cl-doped SnSe_2_: 2SnCl_x_ + 2(2−x)Se → 2SnSe_2−x_Cl_x_, followed by the second annealing process in fused silica tubes at 600 °C for 48 hrs. To improve homogeneity, the synthesized samples were ground into powder form in an agate mortar and re-annealed by repetition of the process described above. Densification of all synthesized samples was conducted with spark plasma sintering (SPS) equipment at 500 °C for 10 min under a pressure of 70 MPa, which resulted in higher densities than 95% of theoretical values.

### Structural and electrical analysis

The crystalline phases of the fabricated samples were identified by PXRD using a Bruker diffractometer model D8 ADVANCE (Cu K_α_). To measure the electrical properties of polycrystalline Cl-doped SnSe_2_ pellets, electrical contacts in the four point-probe and Hall bar geometries were made by applying Ag paste onto the samples. The four point-probe resistivity and Hall coefficient were measured in the temperature range of 6–300 K using Physical Property Measurement System (PPMS, Quantum Design).

### Spectroscopic analysis

The XPS was carried out using focused monochromatized Al K_α_ radiation (1486.6 eV). Raman spectra were measured by confocal Raman spectrometer with 488 nm wavelength excitation.

### Electronic structure calculations

To investigate electronic structure of SnSe_2_, first principle calculations implemented in the Vienna ab initio simulation program (VASP) code were performed. The generalized gradient approximation (GGA) in Perdew-Burke-Ernzerhof (PBE) functional form and the projected augmented plane-wave (PAW) method were employed, which is distinct from previous calculation methods^27^,^28^,^29^. The plane-wave basis-sets with 240 eV cut-off were used. And valence electron states of 4*d*^10^5*s*^2^5*p*^2 ^and 4*s*^2^4*p*^4^ were considered for Sn and Se atoms, respectively. The convergence threshold was set to be 10^−2 ^eV·Å^−1^ and 10^−4 ^eV for force and energy minimization, respectively. The experimental lattice constants of a = 3.81 Å and c = 6.13 Å for SnSe_2_ were used.

## Additional Information

**How to cite this article**: Il Kim, S. *et al*. Metallic conduction induced by direct anion site doping in layered SnSe_2_. *Sci. Rep*. **6**, 19733; doi: 10.1038/srep19733 (2016).

## Supplementary Material

Supplementary Information

## Figures and Tables

**Table 1:** Nominal amount of Cl contents and lattice parameters extracted from PXRD patterns of respective Cl-doped SnSe_2_ samples.

sample	Nominal amount of Cl content	Lattice parameter	
	(x)	a (Å)	c (Å)
A	0	3.81043 (35)	6.13279 (37)
B	0.002	3.81085 (20)	6.13322 (20)
C	0.004	3.81086 (20)	6.13408 (30)
D	0.006	3.81126 (18)	6.13321 (23)
E	0.010	3.81097 (20)	6.13392 (29)
F	0.020	3.81055 (28)	6.13306 (32)
G	0.030	3.80848 (33)	6.13364 (59)
H	0.040	3.80934 (34)	6.13173 (36)

## References

[b1] Novoselov K. S. . A roadmap for graphene. Nature, 490, 192–200 (2012). 23060189 10.1038/nature11458

[b2] Wang Q. H., Kalantar-Zadeh K., Kis A., Coleman J. N. & Strano M. S. Electronic and optoelectronics of two-dimensional transition metal dichalcogenides. Nature Nanotechnol. 7, 699–712 (2012). 23132225 10.1038/nnano.2012.193

[b3] Lee K., Kim S. W., Toda Y., Matsuishi S. & Hosono H. Dicalcium nitride as atwo-dimensional electride with an anionic electron layer. Nature, 494, 336–340 (2013). 23364689 10.1038/nature11812

[b4] Coleman J. N. . Two-dimensional nanosheets produced by liquid exfoliation of layered materials. Science, 331, 568–571 (2011). 21292974 10.1126/science.1194975

[b5] Ye J. T. . Superconducting dome in a gate-tuned band insulator. Science, 338, 1193–1196 (2012). 23197529 10.1126/science.1228006

[b6] Radisavljevic B. & Kis A. Mobility engineering and a metal–insulator transition in monolayer MoS_2_. Nature Mater. 12, 815–820 (2013). 23793161 10.1038/nmat3687

[b7] Khrapach I. . Novel highly conductive and transparent graphene-based conductors. Adv. Mater. 24, 2844–2849 (2012). 22535615 10.1002/adma.201200489 PMC3715101

[b8] Hermann A. M., Somoano R., Hadek V. & Rembaum A. Electrical resistivity of intercalated molybdenum disulfide. Solid State Commun. 13, 1065–1068 (1973).

[b9] Formstone C. A., Kurmoo M., FizGerald E. T., Cox P. A. & O’Hare D. Single-crystal conductivity study of the tin dichalcogenide SnS_2–x_Se_x_ intercalated with cobaltocene. J. Mater. Chem. 1, 51–57 (1991).

[b10] Xia C. . The characteristics of n- and p-type dopants in SnS_2_ monolayer nanosheets. Phys. Chem. Chem. Phys. 16, 19674–19680 (2014). 25111635 10.1039/c4cp02214a

[b11] Palosz B. & Salje E. Lattice parameters and spontaneous strain in *AX*_2_ polytypes: CdI_2_, PbI_2_, SnS_2_ and SnSe_2_. J. Appl. Cryst. 22, 622–623 (1989).

[b12] Atkins P., Overton T., Rourke J., Weller M. & Armstron F. Inorganic Chemistry 5th ed. (Oxford University Press, 2010).

[b13] Nefedov V. I. X-ray photoelectron spectra of halogens in coordination compounds. J. Electron. Spectrosc. 12, 459–476 (1977).

[b14] Mead D. G. & Irwin J. C. Raman spectra of SnS_2_ and SnSe_2_. Solid State Commun. 20, 885–887 (1976).

[b15] Nickel N. H., Lengsfeld P. & Siever I. Raman spectroscopy of heavily doped polycrystalline silicon thin films. Phys. Rev. B 61, 15558–15561 (2000).

[b16] Maciel I. O. . Boron, nitrogen and phosphorous substitutionally doped single-wall carbon nanotubes studied by resonance Raman spectroscopy. Phys. Status Solidi B 246, 2432–2435 (2009).

[b17] Lv R. . Nitrogen-doped graphene: beyond single substitution and enhanced molecular sensing. Sci. Rep. 2, 586; doi: 10.1038/srep00586 (2012). 22905317 PMC3421434

[b18] K. Nomura . Carrier transport in transparent oxide semiconductor with intrinsic structural randomness probed using single-crystalline InGaO_3_(ZnO)_5_ films. Appl. Phys. Lett. 85, 1993–1995 (2004).

[b19] Kasap S. O. Principles of Electronic Materials and Devices 2^nd^ ed. (McGraw-Hill, 2002).

[b20] Gowers J. P. & Lee P. A. Mobility of electrons in SnS_2_ single crystals. Solid State Commun. 8, 1447–1449 (1970).

[b21] Ioffe A. F. & Regel A. R. Progress in Semiconductors, (Heywood, 1960).

[b22] Makise K. . Relationship between variable range hopping transport and carrier density of amorphous In_2_O_3_–10 wt. % ZnO thin films. J. Appl. Phys. 112, 033716 (2012).

[b23] Makise K. . Metal-insulator transitions in IZO, IGZO, and ITZO films. J. Appl. Phys. 116, 153703 (2014).

[b24] Graham M. R., Adkins C. J., Behar H. & Rosenbaum R. Experimental study of the Ioffe-Regal criterion for amorphous indium oxide films. J. Phys.:Condens. Matter 10, 809–819 (1998).

[b25] Pearson G. L. & Bardeen J. Electrical properties of pure silicon and silicon alloys containing boron and phosphorus. Phys. Rev. 75, 865 (1949).

[b26] Rich D. H., Samsavar A., Miller T., Leibsle F. M. & Chiang T. C. Degenerate doping and conduction-band properties of Si studied by synchrotron photoemission of Sb/Si(001). Phys. Rev. B, 40, 3469 (1989). 10.1103/physrevb.40.3469 9992311

[b27] He X. & Shen H. Ab initio calculations of band structure and thermophysical properties of SnS_2_ and SnSe_2_. Physica B, 407, 1146–1152 (2012).

[b28] Murray R. B. & Williams R. H. Band structure and photoemission studies of SnS_2_ and SnSe_2_: II. Theoretical. J. Phys. C: Solid State Phys. 6, 3643–3651 (1973).

[b29] Fong C. Y. & Cohen M. L. Electronic energy-band structure of SnS_2_ and SnSe_2_. Phys. Rev. B, 5, 3095–3101 (1972).

