# Supplementary material for: Metallic conduction induced by direct anion site doping in layered SnSe2
Source: Sci Rep. 2016 Jan 21;6:19733. doi: 10.1038/srep19733 (PMC4726434; doi:10.1038/srep19733)
Supplement: Supplementary Information [file srep19733-s1.pdf]

Supplementary Information

Metallic conduction induced by direct anion site doping in layered $SnSe_2$

Sang Il Kim,[†1] Sungwoo Hwang,[†2] Se Yun Kim,[†2] Woo-Jin Lee,[3] Doh Won Jung,[2] Kyoung-Seok Moon,[2] Hee Jung Park,[4] Young-Jin Cho,[2] Yong-Hee Cho,[3] Jung-Hwa Kim,[3] Dong-Jin Yun,[3] Kyu Hyoung Lee,[5] Intaek Han,[2] Kimoon Lee,[*6] and Yoonchul Sohn[*2]

[1]Department of Materials Science and Engineering, The University of Seoul, Seoul 130-743, South Korea

[2]Materials R&D Center, Samsung Advanced Institute of Technology, Suwon 443-370, Republic of Korea

[3]Platform Technology Laboratory, Samsung Advanced Institute of Technology, Suwon 443-370, Republic of Korea

[4]Department of Advanced Materials Engineering, Daejeon University, Daejeon 300-716, Republic of Korea

[5]Department of Nano Applied Engineering, Kangwon National University, Choonchean 200-701, Republic of Korea

[6]Department of Physics, Kunsan National University, Gunsan 573-701, Republic of Korea

[*]e-mail : kimoon.lee@kunsan.ac.kr, yoonchul.son@samsung.com

[†]These authors contributed equally.

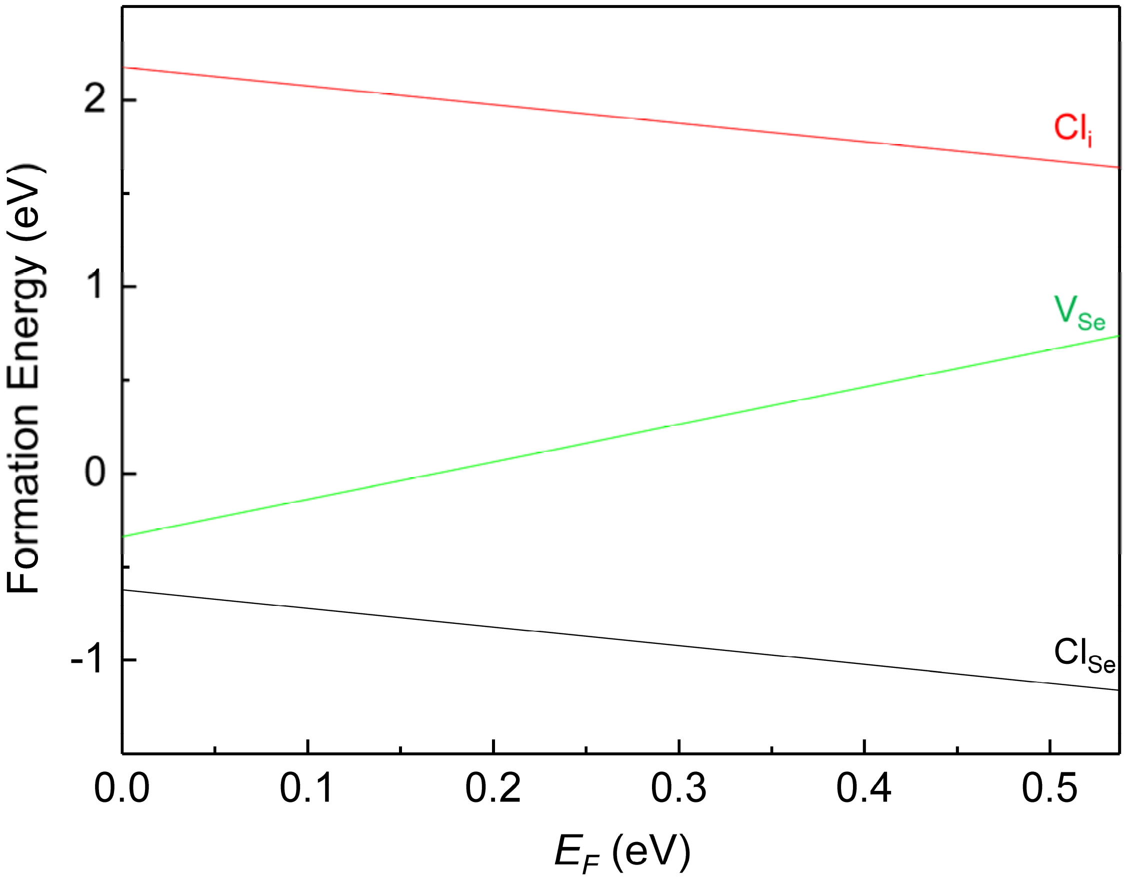

Figure S1. Calculated formation energies for the most probable defect states: Substitutional Cl at Se lattice (Cl_{Se}), Se vacancy (V_{Se}), and interstitial Cl (Cl_i). For the formation energy calculation, we performed the same methods for the band calculation (GGA-PBE-PAW with VASP code) with a supercell containing 72 host atoms (24 Sn and 48 Se atoms) based on a primitive orthorhombic unit cell with one chemical formula.

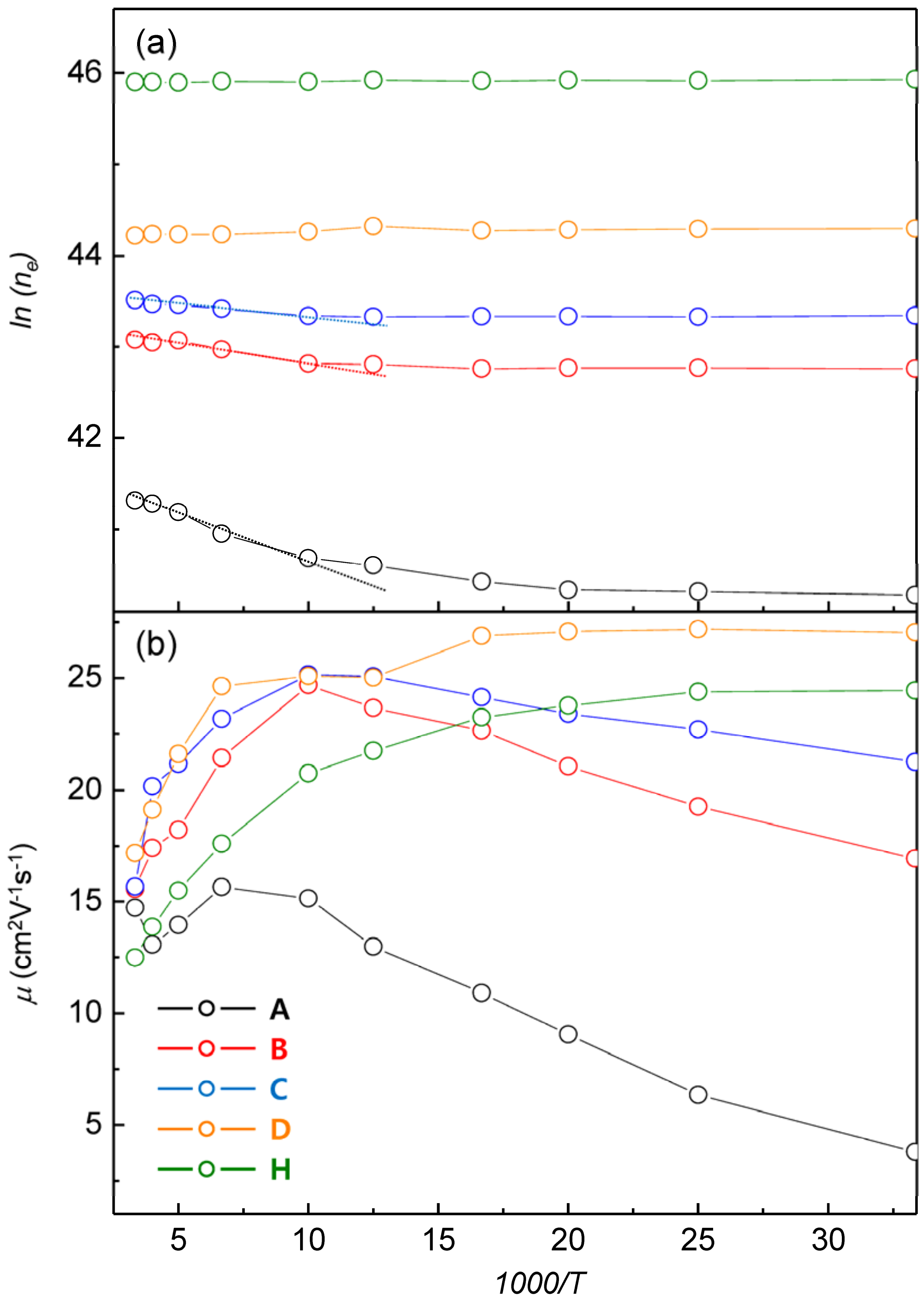

Figure S2. (a) *ln(n$_e$)* and (b) μ vs. *1000/T* plots in high *T* region for Cl-doped $SnSe_2$ samples, where the conduction mechanisms are governed by thermally activated behavior. Dashed lines in Fig. S3(a) guides the linear extrapolation to obtain E_d values.

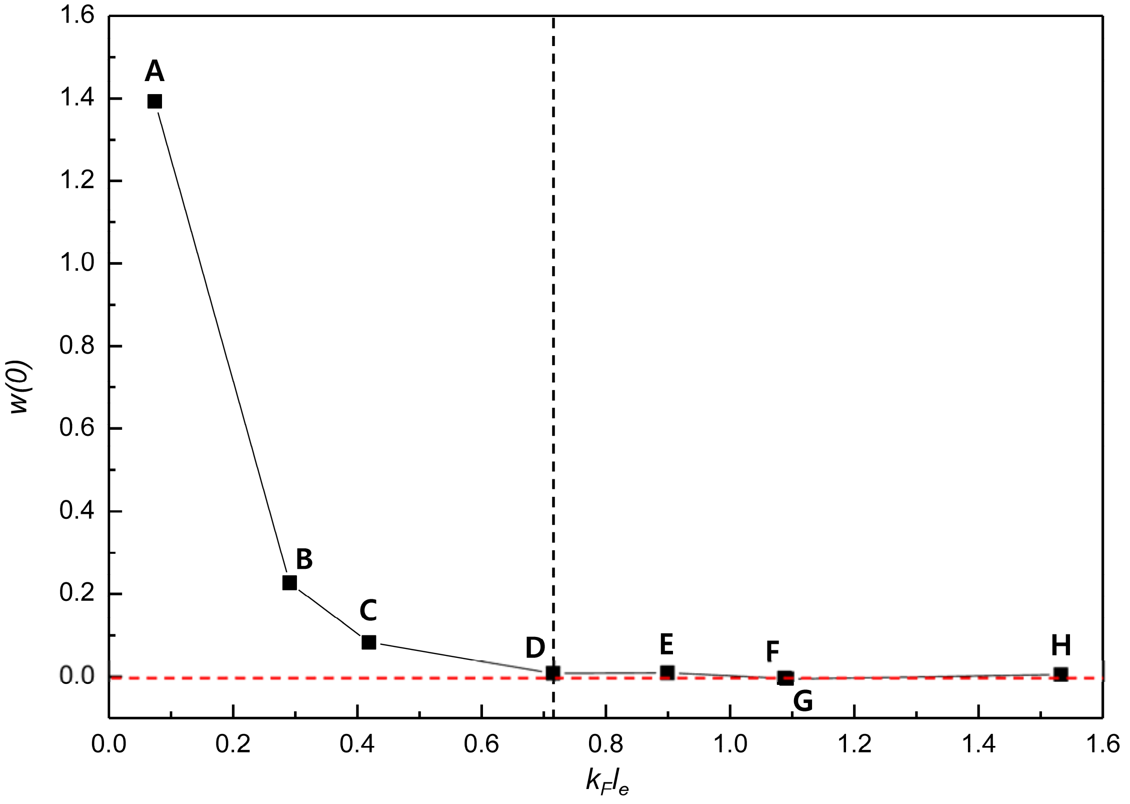

Figure S3. *w(0)* vs. $k_F l_e$ plot to decide $(k_F l_e)_c$ where the metallic conduction emerges by estimating *w(T)* as *T* goes to zero.[M. R. Graham *et al. J. Phys.:Condens. Matter*, **10**, 809 (1998)] The mathematical function of *w(T)* is defined as $d(\ln\sigma)/d(\ln T)$, and *w(0)* should approach zero when the material exhibits the *metallic*. Red and black dashed line guides to the point to be *w(0)* = 0 and $(k_F l_e)_c$, respectively.
